# Another Laurasian connection in the Early Eocene of India: *Myrmecarchaea* spiders (Araneae, Archaeidae)

**DOI:** 10.3897/zookeys.1071.72515

**Published:** 2021-11-17

**Authors:** Hannah M. Wood, Hukam Singh, David A. Grimaldi

**Affiliations:** 1 Department of Entomology, National Museum of Natural History, Smithsonian Institution, Washington, DC 20560, USA National Museum of Natural History, Smithsonian Institution Washington, DC United States of America; 2 Birbal Sahni Institute of Palaeosciences, Lucknow 226007, India Birbal Sahni Institute of Palaeosciences Lucknow India; 3 Division of Invertebrate Zoology, American Museum of Natural History, New York, NY 10024-5192, USA American Museum of Natural History New York United States of America

**Keywords:** Biogeography, exuvium, pelican spider, systematic paleontology, Ypresian

## Abstract

The first fossil Archaeidae in Cambay amber from India, of Eocene age, is documented. The inclusion is a spider exuvium and is placed as *Myrmecarchaea* based on the presence of elongated legs, a slightly elongated pedicel with lateral spurs, and a diastema between coxae III and IV that is similar to *M.antecessor* from Oise amber. The previous occurrences of the genus are from Baltic and Oise amber, both of Eocene age. Because most spiders, including Archaeidae, only molt as juveniles, the exuvium does not have adult features nor have distinct species-specific features, and a new taxon is not erected. This new record further extends the distribution of the family and genus to India 50–52 million years ago. *Myrmecarchaea* in Indian Cambay amber provides additional evidence that India in the Early Eocene had affinities with the Palearctic mainland rather than showing Gondwanan insularity.

## Introduction

Archaeidae Koch & Berendt, 1854 was initially described from fossils in Baltic amber of Eocene age. Decades later, extant species were discovered in the forests of Madagascar (Pickard-Cambridge 1881), and then were also found and documented from South Africa and Australia (Forster and Platnick 1984). The number of extant species continues to grow due to taxonomic revision, some recent (e.g., Lotz 2015; Wood and Scharff 2018). Yet thus far, the extant clades remain known only from these three areas. The fossil record has also expanded, not only in new species, but from new deposits from different parts of the world. Presently archaeid species have been described from the following deposits, ordered chronologically in geological time: Bitterfeld amber, age controversial, but likely middle Eocene (Wolfe et al. 2016; Dunlop et al. 2018); Baltic amber from the “Blue Earth” stratum (which yields much but not all commercial Baltic amber) is of mid-Eocene (Lutetian) age (Ritzkowski 1997); French Oise amber of lower Eocene age (Nel et al. 1999); Burmese amber of Late Cretaceous age (Shi et al. 2012); compression fossils from Inner Mongolia of Late Jurassic age (Huang 2019), and from Kazakhstan dated as Late Jurassic (Doludenko et al. 1990). The fossil record for archaeids is extensive compared to most other spiders, spanning deep geological time and large geographical distances. Many of the archaeid fossils are preserved in amber, which captures exquisite morphological details and thus provides more evidence about evolutionary relationships. While the northern lineages have gone extinct, the southern lineages have persisted, making Archaeidae an intriguing group for understanding ancient biogeography patterns and faunal turnover through deep time.

Herein, we report on the first archaeid documented from Cambay amber, from western India, dated at 50–52 Ma (Rust et al. 2010). The amber piece contains a spider exuvium, and this record extends the known distribution of archaeids to include India. India was once a part of Gondwana and break-up of this landmass started in the Middle Jurassic (Rabinowitz et al. 1983), with India breaking away from Madagascar in the Late Cretaceous (Storey et al. 1995) and traveling northward until colliding with Asia at an age hypothesized to be around 50 Ma (Garzanti et al. 1987; Rowley 1996). Cambay amber documents the Indian biota at a time when it had a tropical, broad-leaved paleoenvironment and around the time of collision with Asia.

## Materials and methods

Fossiliferous amber from the Eocene of India comes from the Cambay and Kutch Basins and is dated as mid- to early-Ypresian (50–52 Ma). The specimen reported here occurs in Cambay amber from the Tadkeshwar lignite mine, approximately 30 km NE of Surat, 21°21.400'N, 073°04.532'E, Gujarat state, India. The stratigraphy of the mines and locations of amber-bearing strata are presented in Rust et al. (2010). The archaeid is the only specimen of the family among the several thousand arthropod inclusions screened thus far in bulk, unprocessed Cambay amber. There is a diversity of other spiders and arachnids in this amber.

**Figure 1.**
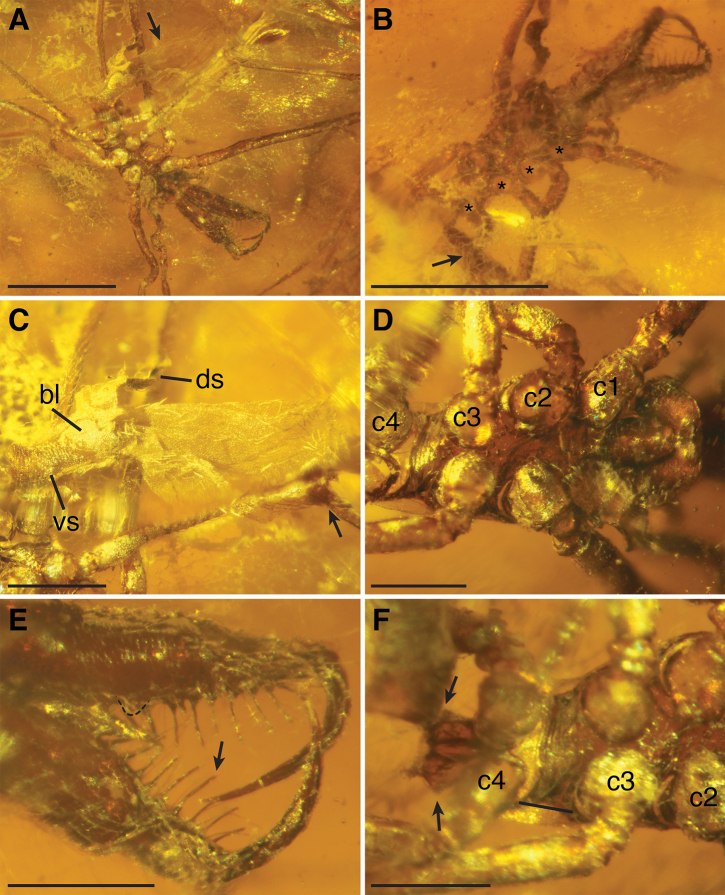
Images of *Myrmecarchaea* sp. (BSIP 41985) from Cambay amber **A** exuvium, ventral; arrow pointing to dorsal of abdomen **B** cephalothorax, dorsal; asterisks denote the coxal openings on the right side where the legs were pulled out of the ventral portion of the exuvium; arrow pointing to some silk threads that is part of a mesh that covers the dorsum of the exuvium **C** abdomen, lateral; arrow pointing at spinnerets; ‘ds’ showing the anterior dorsal abdominal sclerite, which is folded back as part of the molting process when the spider freed its body from the exuvium; ‘bl’ marking the booklung cover that is attached to the anterior ventral abdominal sclerite (labeled ‘vs’) **D** anterior portion of cephalothorax, ventral; for reference, the coxae on the right side are numbered and labeled (c = coxa) **E** distal portion of chelicerae, posterior; dashed line outlines the cheliceral gland mound on the right chelicera; arrow points to one peg tooth **F** posterior portion of cephalothorax, ventral; for reference, the coxae on the left side are numbered and labeled (c = coxa); arrows show the lateral spurs on the pedicel; black line shows the diastema between coxa III and coxa IV. Scale bars: 1 mm (**A, B**); 0.25 mm (**C–F**).

The amber piece contains an archaeid exuvium (Fig. 1). Following Henningsmoen’s criteria for recognizing exuviae (1975), the position of the different pieces of the exuvium and the sutures where the exuvium are broken are in line with what is expected when a spider molts and removes its soft body from the molt. In spiders, first, the cephalothorax breaks laterally, starting near the clypeus and then extending posteriorly until the carapace lifts off; next, the lateral tears extend to the anterior of the abdomen (opisthosoma); lastly, the spider extracts its body out of the lower portion of the exuvium (Foelix 2011). In the majority of spiders, molting only occurs until the adult stage (Foelix 2011), and this has also been observed for archaeids (H.M.W. personal observation). In fact, the cylindrical carapace is fully fused in adult archaeid specimens, completely encircling the cheliceral bases (Wood et al. 2012), and this configuration would likely prevent molting in adult specimens. Thus, the exuvium does not have adult features, but the size of the exuvium, with most adult archaeids being 2–4 mm in size, suggests this may have been the shed skin of a penultimate female that became an adult.

The more sclerotized portions of the exuvium are the chelicerae, sternum, coxae, pedicel, and anterior-most portion of the abdomen, and these structures retain what is probably much of their original pre-molting shape. Some parts of the legs are deformed, containing bends or shriveling, and since most of the abdomen is less sclerotized, it is also deformed. The exuvium has all parts remaining (chelicerae, lower half of the cephalothorax, and abdomen) except for the carapace and some distal parts of the legs. The exuvium is resting on what appears to be a spider web or silk mesh (Fig. 1B). Archaeids do not construct webs for catching prey, and are instead active hunters specialized to prey on other spiders (Millot 1948; Legendre 1961; Wood et al. 2012). But archaeids do construct silk snares and draglines that they hang from while molting (H.M.W. personal observation). This is not likely the case though for the silk observed in this amber piece because the dorsum of the exuvium is resting on the silk rather than the ventral portion, which would be expected during molting. Instead, it could be that after molting the exuvium was carried in the wind or dropped from above and was captured by the web and/or tree resin. There are other unknown, circular bundles, nearby, possibly of debris.

The amber piece was trimmed and polished, then embedded in EpoTek301-2 synthetic resin, followed by additional trimming and polishing. The specimen was observed with a Leica 205C and an Olympus SZX10 microscope. Photographs were taken as a series of stacks using a Canon EOS T6i digital camera mounted to the Leica microscope. Image stacks were assembled into one combined image using ZereneStacker (Zerene Systems, LLC). All measurements are in millimeters (mm).

## Systematic paleontology

**Superfamily Palpimanoidea sensu Wood et al. (2012)**

### Family Archaeidae Koch & Berendt, 1854

#### Genus Myrmecarchaea

Taxon classificationAnimaliaAraneaeArchaeidae

Wunderlich, 2004

2FD08425-FCDE-5603-9E1F-A94E63D9C21F

##### Remarks.

**Figure 2.**
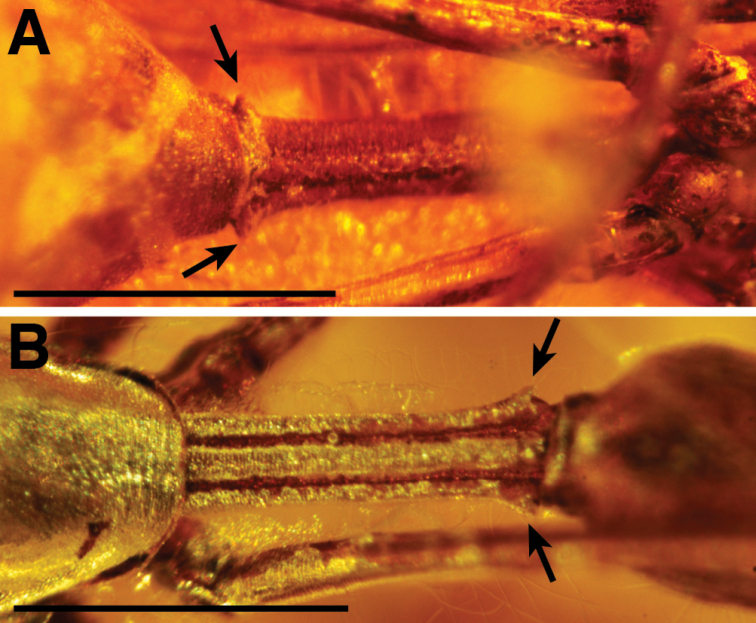
Pedicel of different *Myrmecarchaea* species from Baltic amber, arrows marking lateral spurs **A***M.pediculus* Wunderlich, 2004, pedicel, ventral, holotype specimen, No. S3907/4338, from Geologisch Paläontologisches Institute und Museum (GPIH) **B***M.petiolus* Wunderlich, 2004, pedicel, dorsal, holotype specimen, No. S3999/4337, from GPIH. Scale bars: 0.5 mm.

The presence of a cheliceral gland mound, peg teeth running along the inner cheliceral margin, cuticle texture with scales and/or tubercles (in this case, having both), and the lack of leg spines indicate Palpimanoidea. The following characters indicate Archaeidae: setal bases on tubercles on the sternum, the shape of the sternum (narrow throughout, not shield shaped), the elongated chelicerae, the shape of the gland mound (pointed, positioned close to fang tip), the blunt setae on the abdomen (rather than tapering), the presence of a bump on the dorsal, basal surface of the femora, and the presence of a curve in femur IV. The specimen is referred to as *Myrmecarchaea* based on having a slightly elongated pedicel and greatly elongated legs (Wunderlich 2004). Specifically, elongated legs are defined here as femur I being at least four times as long as the carapace length. Another diagnostic character for the genus may be the presence of a spur on each lateral side of the pedicel, adjacent to the anterior of the abdomen (Fig. 1F). The presence of lateral spurs is also observed in *M.petiolus* Wunderlich, 2004, and *M.pediculus* Wunderlich, 2004 (Fig. 2; pedicel is obscured in the single known specimen of *M.antecessor*Carbuccia et al., 2020). There are other palpimanoid genera with elongated legs, including both extinct (e.g., *Planarchaea* Wunderlich, 2015) and extant members (e.g., *Eriaucheniusworkmani* Pickard-Cambridge, 1881, although with only leg I elongated). However, these other taxa do not also have an elongated pedicel, nor a pedicel with lateral spurs.

*Myrmecarchaea* is comprised of three species: *M.petiolus*, *M.pediculus*, and *M.antecessor*. The exuvium shows similarities to *M.antecessor* in having a diastema between coxae III and IV (compare Fig. 1D, F with fig. 2 from Carbuccia et al. 2020). The pedicel seems slightly longer than in non-*Myrmecarchaea* archaeids, but not as extreme as the pedicel of *M.petiolus* and *M.pediculus*. The ratio of cephalothorax length to pedicel length can be used to compare these shape differences: *M.pediculus* = 1.2; *M.petiolus* = 1.4; *M.antecessor* = 2.3 (estimated from figures in Carbuccia et al. 2020); *E.workmani* = 4.3. This ratio should be treated with caution because measurements were taken from different views for the different species out of necessity due to inconsistencies in fossil preservation. The exuvium from Cambay amber has a ratio of 4.0, and does not present a remarkably long pedicel. The adult ratio may be closer to that of *M.antecessor*, but because this exuvium is from a juvenile, it cannot be determined whether this is *M.antecessor* or a new species.

#### Myrmecarchaea

Taxon classificationAnimaliaAraneaeArchaeidae

sp.

280171E7-9A81-545C-BA8B-16045E220FDB

##### Material examined.

single specimen, voucher number BSIP41985 (collection details above), deposited in Birbal Sahni Institute for Palaeosciences in Lucknow, India.

##### Description.

Body length from endites to abdomen: 2.4 mm, but abdominal portion of exuvium is partially deformed (Fig. 1C). Carapace missing. Chelicerae texture with scales and also tubercles present at setal bases (Fig. 1E). Sternum and chelicerae setae white and thickly plumose. Posterior sternum tubercle absent (Fig. 1F). Sternum not fused to intercoxal sclerites, with thin suture separating the two. Intercoxal sclerites large, filling up the intercoxal space. Sternum length 0.52 mm and width 0.21 mm, narrow throughout (longer than wide) and not shield shaped (Fig. 1D). Pedicel 0.21 mm long and 0.18 mm wide. Spur on each lateral side of pedicel (Fig. 1F). Posterior of cephalothorax elongated with a large space (0.084 mm) between coxae III and IV compared to spaces between other coxae (e.g., 0.048 mm between coxae II and III), roughly twice the length (Fig. 1F). Labium with narrow, v-shaped notch at tip, not fused to sternum. Endite shape slightly convergent, following line of the labium, then converging at distal end around labium (Fig. 1D). Endites elongated to at least half the length of the cephalothorax, pointing downward around 45°, extending beyond the coxae. Patella IV with retrolateral bulge, unclear if present on other patella. Large tubercles absent on legs, leg texture with scales. Femur IV with distinct bend. Dorsal surface of femora with bump. Leg IV patella/tibia juncture straight, not hyperextended. Femur I base the same thickness as other femora (Fig. 1D). Femur I longest (2.05 mm), followed by femur II (1.60 mm), femur IV (1.37 mm), then femur III (1.05 mm). Trochanters entire. Leg spines absent. Chelicerae 0.80 mm long and 0.17 mm wide (at midpoint), anterior surface smooth, i.e., lacking spine, protuberance, or cluster of setae. Basal edge of chelicerae splayed out rather than with parallel edges. Slight constriction at basal edge just distal to splayed edge. 8–9 visible peg teeth present only on cheliceral promargin, peg teeth uneven lengths, not showing a pattern (e.g., short, long, short, long), with blunt tips rather than tapering (Fig. 1E). Longer peg teeth present, close to gland mound, and at least one peg tooth present that is anterior to main promargin row. Four teeth on cheliceral retromargin. Cheliceral stridulatory striae present, occurring in the basal 1/3 of chelicera, with a regular edge forming an oval patch. Stridulatory cusps present on pedipalpal femora, two visible on basal right femur and one on basal left, with distal remainder of femora obscured. Distal portion of chelicerae curved laterad, with distal tip tapering, rather than blunt (Fig. 1E). Cheliceral gland mound present, a pointed bulge on retromargin close to where closed fang tip would meet cuticle (Fig. 1E). Fangs evenly rounded, lacking increased curvature at tip. Abdomen 1.35 mm long, exuvium shape suggests abdomen was smoothly rounded, elongate, and lacks dorsal tubercles. Abdomen hairs thick, plumose, with tips blunt and club-like (Fig. 1C). Anterior lateral, posterior lateral, and posterior median spinnerets developed (Fig. 1C). Large sclerotized pits on abdomen absent. Dorsal and ventral sclerotization on abdomen anterior, forming a sclerotized circle around pedicel, with dorsal sclerite folded back due to molting process (Fig. 1C). Pedipalpal tarsus lacking prolateral and retrolateral brush of setae, and spines.

## Discussion and conclusions

### Taxonomic placement and distribution

The fossil from Cambay amber is the first record of an archaeid from India. *Myrmecarchaea* is comprised of three species and was originally diagnosed based on having an elongated pedicel and elongated legs (Wunderlich 2004). We include an additional diagnostic feature for the genus of having lateral spurs on the posterior of the pedicel (Figs 1F and 2). The distribution of *Myrmecarchaea* is expanded to include the following deposits: Baltic amber, French Oise amber, and Indian Cambay amber. These three deposits are all from the Eocene, with Cambay and Oise amber older, dated Ypresian, and most of the commercially sold Baltic amber containing inclusions, dated Lutetian. The Cambay amber specimen is morphologically the most similar to *M.antecessor* from Oise amber, but is separated from that deposit today by over 6000 km.

The widespread nature of Archaeidae in general, and *Myrmecarchaea* specifically, shows a formerly more widespread distribution. One scenario for widespread distributions is the global hothouse climate in the Paleogene, due to the Paleocene-Eocene Thermal Maximum (PETM) and the Early Eocene Climatic Optimum (EECO) (Pearson et al. 2001; Jahren 2007). Indeed, the Cambay amber was formed in coastal, monsoonal, humid dipterocarp forests around this time, with mangroves close by (Rust et al. 2010). Present-day tropical taxa that occurred in northern latitudes during the Paleogene may have retreated southward with the tropical forests when the Earth cooled from late in the Eocene to the Neogene. Today, extant archaeids are considered microendemics (Rix and Harvey 2012; Wood et al. 2015), often occurring on a single mountain top. The specimen from Cambay amber may be a new, undocumented species or may be *M.antecessor*. Future discovery of more specimens hopefully will resolve this issue.

*Myrmecarchaea* are rare in collections, with species only known from 1 or 2 specimens. Only one adult male has ever been documented, that of *M.antecessor* whose male pedipalps (secondary genitalia) show remarkable similarity to the fossil archaeid *Archaeaparadoxa*: “The general structure of the male palp is . . . very similar to *Archaea* . . . with the same general shape of the palpal bulb, the same orientation and shape, including a spiral of the embolus, and also with tegular apophyses in similar positions” (Carbuccia et al. 2020; compare fig. 3 with fig. 7). *Archaea* is comprised of four species and occurs in Baltic and Bitterfeld amber (Dunlop et al. 2020), and *A.paradoxa* is the only species of the genus where adult male specimens have been documented. While *A.paradoxa* and *M.antecessor* have different somatic features, the morphology of the male pedipalps is conserved. This scenario is similar to what has been observed in the extant Madagascan “workmani-group” and the “vadoni-group”, where genitalic differences are subtle, but non-sexual, somatic features, such as carapace shape and abdomen color, are distinct (Wood and Scharff 2018). The diagnostic features of *Myrmecarchaea* argue for monophyly of the genus, but the conserved genitalia suggest shared common ancestry for *Myrmecarchaea* and *Archaea*. The somatic differences between species in these genera suggest substantial divergence in ecology.

### Natural history and trait evolution

The cephalic area of archaeid spiders is highly modified compared to most other spiders: the carapace is elevated and tubular, and encircles the cheliceral bases, and the chelicerae are greatly elongated. This morphology relates to their specialized behavior of actively searching for and preying on other spiders, and allows the elongated chelicerae to be extended 90° away from the body in order to attack spider prey at a distance (Millot 1948; Legendre 1961; Wood et al. 2012). The degree of elevation in the cephalic area and chelicerae has served as the basis for historical classifications of archaeid spiders and their closest relatives (Legendre 1970; Forster and Platnick 1984). However, it has since been shown that elongation of the cephalic area and chelicerae has evolved independently within the family (Wood et al. 2007). There has also been a shift in elevation of the cephalic area through time: in general, fossil archaeids have less elevated carapaces and chelicerae, occupying a unique region of morphospace, whereas the extant clades have more elevated carapaces and chelicerae (Wood 2017). *Myrmecarchaea* and *Archaea* have relatively shorter carapaces and chelicerae compared to the extant clades, especially those from Madagascar and Australia. The Cambay amber fossil exuvium is missing the carapace, but is likely similar in elevation to the carapaces of other *Myrmecarchaea* and *Archaea* based on its cheliceral structure. Future discovery of additional specimens will provide insight into evolution of carapace and cheliceral shape.

### Biogeography

In Archaeidae, the northern lineages have gone extinct and the southern lineages have persisted, producing a pattern where the extant lineages are confined to the Southern Hemisphere, and fossil lineages are known only from the Northern Hemisphere. Phylogenetic and divergence dating analyses, that include fossils together with extant taxa as terminal tips, suggest distinct northern and southern faunas, and that the split between them is congruent with the timing of Pangaea breaking into Gondwana and Laurasia in the Jurassic (Wood et al. 2013). Along these lines, examination of the spider fossil record revealed that Palpimanoidea, to which Archaeidae belongs, began diversifying in the Mesozoic, and Palpimanoidea and Synspermiata were the dominant spider fauna in the Mesozoic, until faunal turnover in the Cenozoic when they were replaced by Araneoidea and the RTA-clade (Magalhães et al. 2020). Thus, archaeids were at one time more widespread, a more dominant part of the spider fauna, and with diversification patterns showing congruence with the break-up of Pangaea. The discovery of *Myrmecarchaea* in Cambay amber adds another piece of evidence suggesting a distinct Laurasian fauna, specifically with Eocene connections between the Baltic region, Oise, France, and western India.

Among the taxa preserved in Cambay amber that have been studied thus far, some show a Laurasian connection among both living and extinct lineages. The main amber deposits for comparison are the Baltic amber of northern Europe (Lutetian), Oise amber from France (Ypresian), and Fushun amber of northeast China (Ypresian). Laurasian taxa include the following: melikertine bees from both Baltic and Cambay amber (Engel et al. 2013); some long-proboscid fungus gnats (Lygistorrhinidae, Sciaroidea) from both Baltic and Cambay amber (Stebner et al. 2017a); biting midges (Diptera, Ceratopogonidae) from Baltic, Fushun, and Cambay amber, and from the Recent (Stebner et al. 2017b); and termites from Baltic and Cambay amber (Engel et al. 2011a). However, there are a few Cambay amber arthropods showing Gondwanan connections, specifically: a webspinner (Embiodea, Scelembiidae) which occurs today in Africa and South America (Engel et al. 2011b); and a whip spider (Amblypygi, *Paracharonopsis*), apparently closely related to the monotypic, relict African genus *Paracharon* (Engel and Grimaldi 2014). There are even two examples of Cambay amber taxa where each has connections to the Recent and Miocene (Dominican Republic amber) of the Neotropical Region: Leptosaldinae bugs (Heteroptera, Leptopodidae) (Grimaldi et al. 2013b), and some dusky-wing lacewings (Neuroptera, Coniopterygidae, *Spiloconis*) (Grimaldi et al. 2013a). These examples show that the Cambay amber has disparate connections to other regions of the world, signaling widespread affinities. Thus far there is no evidence that, at the time of formation of Cambay amber, the Indian subcontinent was biotically isolated, as might be seen for example in the Recent fauna of Madagascar and Australia. Given the range in the ages that India is thought to have docked with mainland Asia, from the earliest Paleogene to the Miocene based on geophysical scenarios (Zhu et al. 2005; Ali and Aitchison 2008; Najman et al. 2010; White and Lister 2012), the paleontological evidence supports the earlier end of this spectrum, probably Paleocene.

## Supplementary Material

XML Treatment for Myrmecarchaea

XML Treatment for Myrmecarchaea

## References

[B1] Ali JR Aitchison JC (2008) Gondwana to Asia: Plate tectonics, paleogeography and the biological connectivity of the Indian sub-continent from the Middle Jurassic through latest Eocene (166–35 Ma). Earth Science Reviews 88: 145–166. 10.1016/j.earscirev.2008.01.007

[B2] Carbuccia B Wood HM Rollard C Nel A Garrouste R (2020) A new *Myrmecarchaea* (Araneae: Archaeidae) species from Oise amber (earliest Eocene, France). BSGF-Earth Sciences Bulletin 191: 24. 10.1051/bsgf/2020023

[B3] Doludenko M Sakulina G Ponomarenko A (1990) Geologicheskoye stroyenie rayona unikalnogo mestonakhozhdenia posdnejurskoy fauny i flory aule, (Karatau, southern Kazakhstan). Geologicheskii Institut AN SSSR, Moskva.

[B4] Dunlop JA Kotthoff U Hammel JU Ahrens J Harms D (2018) Arachnids in Bitterfeld amber: A unique fauna of fossils from the heart of Europe or simply old friends? Evolutionary Systematics 2: 31–44. 10.3897/evolsyst.2.22581

[B5] Dunlop JA Penney D Jekel D (2020) A summary list of fossil spiders and their relatives. In: World Spider Catalog Natural History Museum Bern. http://wsc.nmbe.ch [version 20.5, accessed on July 19, 2021]

[B6] Engel MS Grimaldi DA (2014) Whipspiders (Arachnida: Amblypygi) in amber from the Early Eocene and mid-Cretaceous, including maternal care. Novitates Paleoentomologicae 9: 1–17. 10.17161/np.v0i9.4765

[B7] Engel MS Grimaldi DA Nascimbene PC Singh H (2011a) The termites of Early Eocene Cambay amber, with the earliest record of the Termitidae (Isoptera). Zookeys 148: 105–123. 10.3897/zookeys.148.1797 PMC3264413 22287892

[B8] Engel MS Grimaldi DA Singh H Nascimbene PC (2011b) Webspinners in Early Eocene amber from western India (Insecta, Embiodea). Zookeys 148: 197–208. 10.3897/zookeys.148.1712 PMC3264408 22287898

[B9] Engel MS Ortega-Blanco J Nascimbene P Singh H (2013) The bees of Early Eocene Cambay amber (Hymenoptera: Apidae). Journal of Melittology 25: 1–12. 10.17161/jom.v0i25.4659

[B10] Foelix RF (2011) Biology of spiders. 3^rd^ edn. Oxford University Press, New York.

[B11] Forster RR Platnick NI (1984) A review of the archaeid spiders and their relatives, with notes on the limits of the superfamily Palpimanoidea (Arachnida, Araneae). Bulletin of the American Museum of Natural History 178: 1–106.

[B12] Garzanti E Baud A Mascle G (1987) Sedimentary record of the northward flight of India and its collision with Eurasia (Ladakh Himalaya, India). Geodinamica Acta 1: 297–312. 10.1080/09853111.1987.11105147

[B13] Grimaldi D Engel MS Singh H (2013a) Coniopterygidae (Neuroptera: Aleuropteryginae) in amber from the Eocene of India and the Miocene of Hispaniola. American museum novitates 2013: 20–39. 10.1206/3770.2

[B14] Grimaldi DA Engel MS Singh H (2013b) Bugs in the biogeography: Leptosaldinae (Heteroptera: Leptopodidae) in amber from the Miocene of Hispaniola and Eocene of India. Journal of the Kansas Entomological Society 86: 226–243. 10.2317/JKES130128.1

[B15] Henningsmoen G (1975) Moulting in trilobites. Fossils and Strata 4: 179–200.

[B16] Huang D (2019) Jurassic integrative stratigraphy and timescale of China. Science China Earth Sciences 62: 223–255. 10.1007/s11430-017-9268-7

[B17] Jahren AH (2007) The Arctic forest of the middle Eocene. Annual Review of Earth and Planetary Sciences 35: 509–540. 10.1146/annurev.earth.35.031306.140125

[B18] Koch CL Berendt GC (1854) Die im Bernstein befindlichen Crustaceen, Myriapoden, Arachniden und Apteren der Vorwelt. In: Berendt GC (Ed.) Die im Bernstein befindlichen organischen Reste der Vorwelt gesammelt in Verbindung mit mehreren bearbeitetet und herausgegeben.Berlin, In Commission der Nicolaischen Buchhandlung, 1–124.

[B19] Legendre R (1961) Études sur les *Archaea* (Aranéides). – II. La capture des proies et la prise de nourriture. Bulletin of the Zoological Society of France 86: 316–319.

[B20] Legendre R (1970) Arachnides-Araignées-Archaeidae. Faune de Madagascar 32: 1–50.

[B21] Lotz LN (2015) A new species of *Afrarchaea* (Araneae: Archaeidae) from South Africa. African Invertebrates 56: 409–414. 10.5733/afin.056.0211

[B22] Magalhães IL Azevedo GH Michalik P Ramírez MJ (2020) The fossil record of spiders revisited: implications for calibrating trees and evidence for a major faunal turnover since the Mesozoic. Biological Reviews 95: 184–217. 10.1111/brv.12559 31713947

[B23] Millot J (1948) Faits nouveaux concernant les *Archaea* (Aranéides). Mémoires L’Institut Scientifique de Madagascar, Série A 1: 3–14.

[B24] Najman Y Appel E Boudagher‐Fadel M Bown P Carter A Garzanti E Godin L Han J Liebke U Oliver G (2010) Timing of India‐Asia collision: Geological, biostratigraphic, and palaeomagnetic constraints. Journal of Geophysical Research: Solid Earth 115: B12416. 10.1029/2010JB007673

[B25] Nel A de Plöeg G Dejax J Dutheil D de Franceschi D Gheerbrant E Godinot M Hervet S Menier J-J Augé M (1999) An exceptional Sparnacian locality with plants, arthropods and vertebrates (Earliest Eocene, MP7): le Quesnoy (Oise, France). Comptes Rendus de l’Academie des Sciences Series IIA Earth and Planetary Science 1: 65–72. 10.1016/S1251-8050(99)80229-8

[B26] Pearson PN Ditchfield PW Singano J Harcourt-Brown KG Nicholas CJ Olsson RK Shackleton NJ Hall MA (2001) Warm tropical sea surface temperatures in the Late Cretaceous and Eocene epochs. Nature 413: 481–487. 10.1038/35097000 11586350

[B27] Pickard-Cambridge O (1881) On some new genera and species of Araneidea. Proceedings of the Zoological Society of London 49: 765–775. 10.1111/j.1096-3642.1881.tb01333.x

[B28] Rabinowitz PD Coffin MF Falvey D (1983) The separation of Madagascar and Africa. Science 220: 67–69. 10.1126/science.220.4592.67 17736163

[B29] Ritzkowski S (1997) K-ar-altersbestimmungen der bernsteinführenden sedimente des samlandes (paläogen, bezirk kaliningrad). Metalla (Sonderheft) 66: 19–23.

[B30] Rix MG Harvey MS (2012) Phylogeny and historical biogeography of ancient assassin spiders (Araneae: Archaeidae) in the Australian mesic zone: evidence for Miocene speciation within Tertiary refugia. Molecular Phylogenetics and Evolution 62: 375–396. 10.1016/j.ympev.2011.10.009 22040763

[B31] Rowley DB (1996) Age of initiation of collision between India and Asia: A review of stratigraphic data. Earth and Planetary Science Letters 145: 1–13. 10.1016/S0012-821X(96)00201-4

[B32] Rust J Singh H Rana RS McCann T Singh L Anderson K Sarkar N Nascimbene PC Stebner F Thomas JC Solórzano Kraemer M Williams CJ Engel MS Sahni A Grimaldi D (2010) Biogeographic and evolutionary implications of a diverse paleobiota in amber from the early Eocene of India. Proceedings of the National Academy of Sciences 107: 18360–18365. 10.1073/pnas.1007407107 PMC2972964 20974929

[B33] Shi G Grimaldi DA Harlow GE Wang J Wang J Yang M Lei W Li Q Li X (2012) Age constraint on Burmese amber based on U–Pb dating of zircons. Cretaceous Research 37: 155–163. 10.1016/j.cretres.2012.03.014

[B34] Stebner F Singh H Rust J Grimaldi DA (2017a) Lygistorrhinidae (Diptera: Bibionomorpha: Sciaroidea) in early Eocene Cambay amber. PeerJ 5: e3313. 10.7717/peerj.3313 PMC5437858 28533964

[B35] Stebner F Szadziewski R Singh H Gunkel S Rust J (2017b) Biting midges (Diptera: Ceratopogonidae) from Cambay amber indicate that the Eocene fauna of the Indian subcontinent was not isolated. PLoS ONE 12: e0169144. 10.1371/journal.pone.0169144 PMC5226682 28076427

[B36] Storey M Mahoney JJ Saunders AD Duncan RA Kelley SP Coffin MF (1995) Timing of hot spot—related volcanism and the breakup of Madagascar and India. Science 267: 852–855. 10.1126/science.267.5199.852 17813912

[B37] White LT Lister GS (2012) The collision of India with Asia. Journal of Geodynamics 56: 7–17. 10.1016/j.jog.2011.06.006

[B38] Wolfe AP McKellar RC Tappert R Sodhi RN Muehlenbachs K (2016) Bitterfeld amber is not Baltic amber: Three geochemical tests and further constraints on the botanical affinities of succinite. Review of Palaeobotany and Palynology 225: 21–32. 10.1016/j.revpalbo.2015.11.002

[B39] Wood HM (2017) Integrating fossil and extant lineages: an examination of morphological space through time (Araneae: Archaeidae). The Journal of Arachnology 45: 20–29. 10.1636/JoA-S-16-039.1

[B40] Wood HM Gillespie RG Griswold CE Wainwright PC (2015) Why is Madagascar special? The extraordinarily slow evolution of pelican spiders (Araneae, Archaeidae). Evolution 69: 462–481. 10.1111/evo.12578 25491087

[B41] Wood HM Griswold CE Gillespie RG (2012) Phylogenetic placement of pelican spiders (Archaeidae, Araneae), with insight into evolution of the “neck” and predatory behaviours of the superfamily Palpimanoidea. Cladistics 28: 598–626. 10.1111/j.1096-0031.2012.00411.x 34856731

[B42] Wood HM Griswold CE Spicer GS (2007) Phylogenetic relationships within an endemic group of Malagasy ‘assassin spiders’ (Araneae, Archaeidae): ancestral character reconstruction, convergent evolution and biogeography. Molecular Phylogenetics and Evolution 45: 612–619. 10.1016/j.ympev.2007.07.012 17869131

[B43] Wood HM Matzke NJ Gillespie RG Griswold CE (2013) Treating fossils as terminal taxa in divergence time estimation reveals ancient vicariance patterns in the palpimanoid spiders. Systematic Biology 62: 264–284. 10.1093/sysbio/sys092 23192838

[B44] Wood HM Scharff N (2018) A review of the Madagascan pelican spiders of the genera *Eriauchenius* O. Pickard-Cambridge, 1881 and *Madagascarchaea* gen. n. (Araneae, Archaeidae). Zookeys 727: 1–96. 10.3897/zookeys.727.20222 PMC5799789 29416388

[B45] Wunderlich J (2004) Fossil and extant spiders (Araneae) of the superfamily Eresoidea s.l., with special reference to the Archaeidae and remarks on some higher taxa of the superfamily Araneoidea. In: Wunderlich J (Ed.) Beiträge zur Araneologie, 747–808.

[B46] Wunderlich J (2015) On the evolution and the classification of spiders, the Mesozoic spider faunas, and descriptions of new Cretaceous taxa mainly in amber from Myanmar (Burma). In: Wunderlich J (Ed.) .Beiträge zur Araneologie, 21–408.

[B47] Zhu B Kidd WS Rowley DB Currie BS Shafique N (2005) Age of initiation of the India-Asia collision in the east-central Himalaya. The Journal of Geology 113: 265–285. 10.1086/428805

